# A place of many names: how three generations of Bedouin women express the meaning of home

**DOI:** 10.1007/s10901-017-9546-5

**Published:** 2017-04-24

**Authors:** Nuzha Allassad Alhuzail

**Affiliations:** Department of Multidisciplinary Studies, School of Social Work, Sapir Academic College, 7916500 D.N. Hof Ashkelon, Israel

**Keywords:** Home, Bedouin society, Bedouin women, Social change, A transitional society

## Abstract

This article examines how three generations of Bedouin women in southern Israel express what home means to them through the names they give it: *bayt*, *maskan*, and *dar*. Home has always been significant in the lives of Bedouin women, but Bedouin society is undergoing major changes—culturally, socially, and in the form of settlement. The external form of the Bedouin home has changed, too, from a tent to a stone house, from an open structure to a closed one, from being part of the open space of the desert to being a limited space in a neighborhood. To understand the changing meaning of home for Bedouin women during this transition, I conducted a narrative study with 30 women, of three generations that correspond to three periods of settlement, paying particular attention to the names with which they referred to their home. In the nomadic period, the tent, called *bayt*, allowed life to flow between the home and the tribe, providing a sense of security and control over the social environment. In the “sayag” (restriction) period, home was called *maskan*, a place that generates an atmosphere of tranquility and partial control but also distances the women from an external environment that has become unfamiliar and dangerous for them. In the third period, the home, called *dar*, is permanent and more private, but belongs to the husband only. Paradoxically, it provides physical protection but not a sense of security, and it cuts off the women from the external environment.

## Introduction

This article examines how Bedouin women in southern Israel express what home means to them through the words they use to refer to it. Specifically, I look at the names used by three generations of women in three different periods: *bayt*, *maskan*, and *dar* (Omer 2010). Home is a multidimensional institution in terms of time, place, and social relations. It is seen simultaneously as a starting point and a destination, and its meaning is influenced by both past and present conceptions. Therefore, studies about the home relate to it as a journey between the past and the future. These studies also view identity as an important key in the construction of the idea of the home in a context in which people have been moved by force (Kabachnik et al. 2010), as Israel’s Bedouins have: Their identity remains anchored in their old home, even years after the move.

Bedouin women, too, move between the past and the present through their perception of their home. The first generation called its tent home *bayt*, whereas women of the second generation gave the name *maskan* to the home built of temporary materials by the husband. *Maskan* connotes a place that engenders tranquility and spiritual comfort. In contrast, the young women of the third generation call their home *dar*, the equivalent of the English word “house.” This is a modern home built and owned by the husband, reflecting materialism and status and providing privacy, but cutting off women from the external environment and society. Although this is a permanent house, it does not afford the young women a sense of security and control.

The concept of the home space has many meanings (Moore 2000): a locus of permanence, a space that organizes interpersonal relations, a place that creates order in the individual’s experience of the environment, and a safe space from which one can look out (Douglas 1991). The concept of home also relates to people’s internal life (Matari 2005): as a place that satisfies human needs throughout the life cycle; a focus of social activity that allows a person to fulfill individual and social roles; and a place that creates and provides personal and social values (Annison 2000). For the individual, the home provides the basis for self-construction through relations with family members and other significant figures. More broadly, the home is part of a neighborhood and community (Dam and Eyles 2012). The home is also a symbol of the state—the national home on the cultural-ideological level. Those who argue that the home is a feminine sphere maintain that women’s roles as mothers and sustainers of the home distance them from the public sphere (Hewlett 1986; Després 1991).

Women tend to ascribe positive meanings to the home, seeing in it a source of love, affection, intimacy, independence, and power. The home is linked to their personal identity and to important sets of relationships (Saunders 1989). But some studies show the home as symbolizing the burden placed on women’s shoulders, manifested in daily chores, such as cleaning and cooking, and in educating the children and seeing to the needs of the spouse. The home can also symbolize a place of suffering, a limiting and depriving environment in which women sometimes suffer violence (Shalhoub-Kevorkian 2007; Hwang et al. 2011).

In Bedouin society, home is traditionally a place for welcoming guests, providing refuge for those in need, developing intimacy, and creating the family unit, which is the main component of a strong and renowned tribe (Ben-David 2000). For Bedouin women, home has many additional meanings that are described in the research about them. The older participants in the study, who are members of the first generation, describe the tent home as something women created and that showcased their talent and individuality. It also reflected their ability to control the size of the home, its boundaries, and who would enter it. The women protected its contents, and in it they educated the younger generations (Allassad Alhuzaeel 2009). This home was considered a sphere that provided security, warmth, and love for all the generations.

In Bedouin society the home also symbolizes continuity and holding fast to the land, thus validating both the Bedouins’ ownership of the land and their being part of the Arab-Palestinian people, which is engaged in a continuing conflict with the state (Yiftachel 2000; Shalhoub-Kevorkian 2007). Bedouin women view the state’s demolition of Bedouin homes in the south of the country as a policy aimed at harming them personally, socially, and nationally (Allassad Alhuzail 2014). The demolition of homes inflicts emotional harm on all members of the Bedouin community, but particularly on the women, because the home is the physical realization of the human spirit, the extension of the individual’s identity (Shalhoub-Kevorkian 2007; Aburabia-Queder 2011).

Thus, in a discussion of the meaning of home in the lives of Bedouin women, one cannot ignore the changes in their status, in the social structure of Bedouin society, and in the forms of housing—changes that have influenced the women’s perception of home and the meanings they have ascribed to it in each generation and in each period, as reflected in the names they have given it.

The changes in the way of life deriving from the type of settlement and the changes in the lives and status of Bedouin women in the wake of modernization are intertwined (Hijab 2001; Jad 2006). The changes in the living space, the type of settlement, and the range of mobility have influenced the individual perceptions and the meanings that the women have ascribed to the changes in their lives.

Section 2 provides background information on Bedouin society in Israel, briefly outlining the changes in the society and in the form of habitation in three distinct periods. It also describes my focus on intergenerational cultural transmission. Section 3 describes the methodology, and Sect. 4 presents my findings. In Sect. 5 I discuss the findings, and in Sect. 6 I present a summary and my conclusions.

## Background

In recent years, Bedouin society in southern Israel has undergone great social and cultural changes. The process may be divided into three periods according to settlement type: the nomadic period, the period of restriction, and the period of permanent settlement (Al-Atawna 2002; Levinson and Abu Saad 2004).

During the nomadic period (for the purposes of this study, 1938–1950), the Bedouins had a unique lifestyle, and their social structure was based on a complex, heterogeneous, and hierarchical tribal division (Allassad Alhuzail 2013). Nomadic women enjoyed some autonomy: They could buy and sell within their living space (Tal 1995). There was no strict separation between them and the men, and they had covert influence over the management of the nuclear family and even the extended family. Women enjoyed great mobility in the open desert, moving in groups to bring water for the tribe from distant wells (Allassad Alhuzaeel 2009).

The establishment of the State of Israel was an important turning point. In the early 1950s, Israel transferred the 11,000 Bedouins then in the Negev desert to a restricted area in the northern Negev called *sayag*, and they were forbidden to hunt outside it (Meir and Ben-David 1996). The men stayed home more than in the past and assumed some of the women’s roles (Dinero 1997). Women were more strictly guarded because they were more exposed to other tribes. Their mobility was restricted, their roles were limited to household maintenance, and their status in the family dropped. From the 1970s, the process of settling the Bedouins gained momentum as a means of controlling them (Dinero 1997), but also of helping them to adapt to the modern state (Yiftachel 2009).

The transition to permanent settlement had different but decisive consequences for each generation of Bedouin women. The women of the first generation, born into and used to a nomadic life, lost many of their traditional roles as modern appliances took over many of their old tasks. Their forced inactivity affected their degree of control over their families, their ability to instill traditional values, and their child rearing (Al-Krenawi 1996). Second- generation women who lived in both the period of restriction and the period of permanent settlement had to cope with fundamental geographical and social changes. The third generation, born and raised in permanent communities, is trying to integrate rapidly into the world outside the tribes. Each year the number of young women who graduate from high school and receive college degrees increases, as does the number of women who work outside the home and help to support their families. Meanwhile, the traditional support network from which women once drew their power has become less extensive and influential than it was during the nomadic period (Abu-Asba 2008).

### Three periods, three generations, and cultural transmission

The emphasis in this study is on cultural transmission in a traditional society that has undergone change and not on the psychological aspects of the transmission from mother to daughter. Cultural transmission and intergenerational relations in ethnic communities play a significant role in terms of culture and heritage and raise the question of whether, in a context of change, intergenerational transmission enables cultural continuity (Shahar 2008).

This study focuses on how Bedouin women have experienced the changes in their society during three periods that are characterized by different types of settlement and a change of region. Each period corresponds roughly to one generation. Therefore, I chose to study the life stories of three-generation triads within families. The stories revealed the elements that maintained stability in the women’s lives despite the contextual changes.

Intergenerational (parent-child) transmission ranges from precise transmission, with tiny differences, to failed transmission. Both poles are problematic: Perfect transmission does not allow for change and renewal, and total failure does not allow for coordinated action between the generations (Shahar 2008). These issues are generally discussed in the literature on native populations and migrant populations, that is, societies in which exposure to cultural changes leads to intergenerational cultural gaps (Shahar 2008). In any case, in the process of intergenerational transmission, the family is the connecting link (Olson 1993).

## Methodology

The most suitable approach for research that aims to describe holistically that which cannot be observed directly in phenomena, processes, and subjective interpretations is the qualitative paradigm, in accordance with the phenomenological tradition. It makes possible the positing of hypotheses regarding the relations between the meanings of the inter-subjective phenomena, within boundaries set in advance by the researcher (Patton 1987; Miller and Crabtree 1992; Weiss 1994; Lieblich et al. 1995).

The phenomenological assumption here was that changes in the women’s physical environment affect their lives and may change their worldview, while, at the same time, their cultural worldview may shape the meaning that they ascribe to these changes (Allassad Alhuzail 2014). Therefore, the life-story interview seemed most appropriate.

### Participants

The snowball method was used to find participants, starting with a request of the welfare department in Rahat Municipality for help in finding a family that would agree to be interviewed. Each family interviewed referred me to another family until I had ten families unrelated by kinship ties, living in communities whose names are not reported here to protect the identity of the participants (Hodnett 1988; Allassad Alhuzaeel 2009).

The interviewees were 30 Bedouin women, of three generations. In each family, the grandmother decided which of her daughters should be interviewed. In keeping with Bedouin notions of honor (Aburabia-Queder 2008), this was almost always the oldest daughter; only if she refused was the next-oldest daughter chosen. All the women of the second generation directed me to their oldest daughters. I, a Bedouin woman familiar with Bedouin society, translated the interviews into English with the assistance of an English-language editor.

Table 1 shows several clear intergenerational changes. The first is the transition from the tent, in the first generation, to the iron-sheet cabin in the second, and the stone home in the third. The titles that the women have given their life stories also reveal substantial differences: In the first generation, six of the ten women use the plural form of self-reference in their titles, for example, “ours,” “we,” and “women.” In the third generation one sees the women relating to themselves more in the first person. In the first and second generations the women are named Um (“mother of”) their firstborn son, whereas in the third generation the women choose to call themselves by their own first names, though they all have sons.

**Table 1:** Details of the participants, Bedouin women of three generations

First generation	Age	Years of education	Employed	Polygamy	Type of home	Number of children	Title of life story
Um Abdullah	71	0	No	Yes	Tent	10	Our Story
Um Salem	75	0	No	Yes	Tent	8	Our Life
Um Mahmad	70	0	No	No	House	7	The Bedouin Women
Um Diab	69	0	No	Yes	Tent	12	My Life Story
Um El-Abed	80	0	No	No	Tent	12	Life Back Then
Um Sliman	73	0	No	No	Tent	8	A Woman’s Life
Um Ali	71	0	No	Yes	Tent	9	Our Life
Um Wadha	75	0	No	Yes	Tent	11	Life in the Past
Um Fa’iz	70	0	No	No	Tent	8	We, the Bedouin Women
Um Mahmoud	74	0	No	No	Tent	9	The Bedouins Then
Second generation	Age	Years of Education	Employed	Polygamy	Type of home	Number of children	Title of life story
Um Daham	55	3	No	No	Cabin	8	The Beautiful Life
Wahiba	51	5	Yes	No	Cabin	11	The Story of the Day
Na’ama	53	8	No	No	Cabin	8	The Forgotten Woman
Um Wadad	54	3	No	No	Cabin	12	A Woman’s Story
Um Ashraf	50	2	No	No	Cabin	9	There Was a Woman
Um Mahmad	51	6	No	No	Cabin	10	The World of the Bedouins
Um Bassem	56	4	No	No	Cabin	8	A Strong Woman
Um Khaled	53	4	No	No	Cabin	10	Untitled
Um Amar	51	2	No	No	Cabin	8	Once upon a Time
Um Sami	50	3	No	No	Cabin	12	The Bedouins Today
Third generation	Age	Years of Education	Employed	Polygamy	Type of Home	Number of Children	Title of Life Story
Wadha	35	8	No	No	Stone	5	Untitled
Siham	32	8	No	No	Stone	4	Confusion, Lost
Lola	30	12	Yes	No	Stone	4	The Struggle
Hiam	31	12	Yes	No	Stone	6	One Woman’s Story
Shifa	37	12	Yes	No	Stone	1	My Story
Hanin	30	12	Yes	No	Stone	2	The Wisdom of the Present
Halud	33	8	No	No	Stone	7	Quiet
Layla	30	8	No	No	Stone	7	One Woman and the World
Yasmin	33	12	Yes	No	Stone	5	Where Am I?
Ulla	31	8	No	No	Stone	4	God Forbid

The order of the women in the list in each generation reflects her family affiliation: The first woman in the list of the second-generation women is the daughter of the first woman in the list of first-generation women, and so on

With regard to education and employment, it is clear that the women of the first generation are both uneducated and unemployed outside the home. In the second generation the women have no more than a few years’ education, and most are unemployed outside the home. In the third generation there is a clear dichotomy between those who have 8 years’ schooling and are unemployed and those who completed high school and work outside the home. Most of the women have many children, and there are no significant changes between the generations in this respect. With regard to polygamy, half of the first-generation women live in polygamous marriages, whereas none of the women in the second and third generations does (Table 2).

**Table 2:** How Bedouin women of three generations perceive and refer to their homes

Generation	Women’s name for the home	Materials used	Created by	Control over the size	Ownership	Status of the home	Legal status of the settlement	Degree of the home’s openness
First (grandmothers)	*bayt*	Goat hair	The wife	The wife	The wife	Partially nomadic	Unrecognized; traditional ownership of the land	Open on all sides; no door; connected to the environment
Second (daughters)	A place of tranquility: *maskan*	Iron-sheet	The husband	The husband	The husband	Temporary	Unrecognized; state ownership of the land	Totally enclosed; has a door; flexible with regard to the environment
Third (granddaughters)	A permanent and protective home: *dar*	Cement blocks	The husband	The husband	The husband	Permanent	Recognized; private ownership by the husband of land he has bought	Closed, locked, and secure; not flexible with regard to the environment

### Research tools

I used the life-story interview, an open-ended tool that enables one to elicit as rich a narrative as possible regarding the interviewee’s life (Bruner 1986; Patton 1987; McAdams 1988; Gergen 1992; Atkinson 1998). The individual describes the course of his or her life as a collection of events and experiences, in the order in which he or she chooses to tell them (Yehezkel-Friedler 1999). The order and the content reflect the meaning that the events have in the life and identity of the individual (Fischer 1982).

### Data analysis

My aim was to decipher the meaning that the participants ascribe to their home and to their experiences of historical and personal events, whether their involvement was direct or indirect, and to understand how this meaning is reflected in the names the women use to refer to the home (Bruner 1986; Corradi 1991). The research question dealt with experience; therefore, I applied an interpretive analysis to meaning units (Strauss and Corbin 1990; Tutty et al. 1996). The analysis had four stages: reading the life story as a whole text, coding the meaning units, classifying the meaning units by category and theme, and connecting the themes into a whole picture, both for each generation separately and with regard to the intergenerational transitions.

#### Reading the life story as a whole text

In the first stage of the analysis, I read each interview as a whole text in an attempt to identify the main contents reflected in it and their structure (Lieblich et al. 1998). At this stage I acted in accordance with the principle of the researcher who examines the data (in this case, the interviews) repeatedly, until she feels she is familiar with the relevant material and until glimmers of findings begin to appear (Moustakas 1994; Creswell 1998). In the second stage, I categorized the contents by meaning units, using the approach of Tutty et al. (1996), who propose a structured, step-by-step method of analysis, as follows:

#### Coding the meaning units

In each interview I identified words, phrases, and sentences containing specific content, such as “home [*bayt*] but not a house [*maskan*]” “we,” “respect for the home,” and “the family home.” I examined the characteristics of each meaning unit: how the women use it, how it appears in each generation, and in what context it appears. At this stage, I gave free reign to my intuition and imagination in an attempt to approach the research topic from every possible angle. From these meaning units I selected those that are relevant to the research question (Tesch 1990; Moustakas 1994).

#### Grouping the meaning units into categories and themes

I grouped meaning units into categories and themes according to the similarity of their content or structure. A central theme in each of the three generations was the home: (1) home as women’s creation (*bayt*), (2) the family home (*maskan*), and (3) the physical home (*dar*).

#### Connecting the themes

In the fourth stage, I connected the themes to compose a whole picture in a process that involved focusing, comparing, and contrasting the various themes. These conceptualizations explain why people act as they do (Le Compte and Preissle 1994). At this stage it is possible to find a unifying pattern, typologies, or theoretical concepts (Glaser and Strauss 1967; Tesch 1990).

## Findings

The following table describes the home in each generation and how the women perceive and refer to it.

### The rooted home

#### Home (bayt) as women’s creation and under their control

All the parts of this home were woven by the unmarried woman; she determined its size and boundaries. Built on land over which Bedouins have traditional ownership, at a time when Bedouins enjoyed autonomy, the home was open on all sides and connected to the social environment. Women had control within it—educating the children—and also indirect control of tribal matters through the husband. The women call this home *bayt* (in Arabic, a place where one customarily stays and spends the night). They are emotionally attached to it and express powerful longing for this home that no longer exists.

In the nomadic period, women gave birth at home and controlled the birth process, as Um Abdullah says: “*I gave birth to my sons in the home; our lives were better then. The woman who has given birth sits in her house and all around her are women who take care of her, God helps her, her friend is next to her*.”

Unlike Bedouin women describing their lives today, Um Abdullah generally speaks about herself in the third person. This, along with the use of the first-person plural among Bedouin women of her generation, expresses generalization or distancing. Here, Um Abdullah means that in those days all the women behaved this way. And despite the material poverty then, the words give the impression of lively communal relations and a sense of pleasure in the life in which the home is the center. In the life stories of the women, the tent that is open on all sides is the home that provides shelter, protection, and privacy. In this era, when the Bedouin migrated in search of pasture and water, the home provided a sense of permanence. The open home also allowed sensory contact with the other members of the community.

In summary, although the nomadic way of life and the home appear to be temporary, the tent-home was of central importance in the women’s lives. They wove it and within it they wove their lives. The special meaning the home had in their day was anchored in the tent’s flexible boundaries, which allowed life to flow in and out. The cloth partitions allowed information to seep from the men’s section to the women’s. Hospitality, one of the main customs in nomadic life, gave the women an opportunity to display their talents without appearing before the guest. Finally, there was meaning to the connection between the home and the land, the most important thing to Bedouins in all periods. The nomadic period was rich in terms of control of land and the building of homes woven of the hair of the many goats the Bedouins had then.

### The transitional home

#### Home as longing for peace and quiet (maskan)

The home that the second-generation women refer to as *maskan* was built of simple, temporary materials on land that was expropriated from the Bedouin community and now belongs to the state during a traumatic transition from nomadic life to a settled one in a restricted area. Houses were built without permits in villages that are not recognized by the state and consequently are under constant threat of demolition. In this period, the women related to the home as a refuge, providing tranquility and calm in a sea of uncertainty. They refer to it as *maskan*, or *sakina*, words taken from the Quran, meaning a place to which one flees for personal peace and security. Citing a passage from the Quran, the women compare their home to conjugal life that generates calm and tranquility for the couple. Therefore, they see the simple, temporary home as a protective place in which they can find spiritual calm and a connection to Allah, the source of their strength.

The importance of this home is apparent in the words of Um Ashraf: “*Without a home she isn’t worth anything…A woman must, must, must have a house because [without it] she is* oura*, imperfect…If she walks in the night perhaps someone will harm her*.” The emphasis and the repetition of the word “must” express the great importance that Um Ashraf ascribes to the home. She is actually saying that a woman without a home is exposed to harm. This feeling, which was almost totally absent among the first-generation women and is less prominent in the third generation, apparently derives from the change in the form of settlement and the life of the Bedouins during the *sayag* period.

Though the second-generation women sometimes express their longing for the tent home (*bayt*), when they talk about home they mean mainly the nuclear family and the relations between its members. The home is the framework, albeit simple, that unites all the family members. “*In this house* [maskan] *we come together as a family; the husband comes home from work, the children gather, and the joyful voices rise; within these walls the family breathes, lives, and grows*” (Wahiba).

Women had an important role in the home and in the relationships within it, and they could destroy everything built or preserve it, as Na’ama says: “*The woman could destroy her home with her hands. There would be a divorce,…it would destroy the home, the home would be disbanded.*” Although Wahiba agrees with Na’ama, she says that not everything is in the woman’s hands. It is difficult to sustain a home when the husband is weak: “*The home also exists thanks to a strong man. If the man is weak, the children will not respect him and then the home will be destroyed.*”

From Na’ama and Wahiba’s words one can learn how the second-generation women view the good Bedouin woman. One of her main roles is to give support to her husband, and this role is of extreme importance for maintaining the integrity of the home—that is, the family. The fact that the second-generation women draw a parallel between the home and the family and rarely mention the home as a physical structure can be explained by the changes in Bedouin society during the period of the *sayag* when women lost their role of physically creating the home.

In summary, the second generation of women (the mothers) experienced both a significant loss and an important gain. They lost their central role in weaving and planning the tent, along with the sensory connection to the community. In the new, iron-sheet home, this sensory connection to the community was blocked. Most of the day was spent in idleness. On the other hand, women gained technology that made their lives easier and acquired religious knowledge that helped fill the vacuum created by the change.

### The material home

#### A permanent home that is really only temporary

In the third generation, the home belongs to the husband and is built on land bought by him. It fulfills material needs but not emotional ones, and this home that is physically permanent is temporary in the religious sense. Consequently, the women call it *dar* (in Arabic, “the locus of family activity,” but here meaning simply “house”) and sometimes *dar el*-*dunya* (a phrase inspired by the Quran meaning “the earthly home,” in contrast to the true permanent home—*dar el*-*ahra*—in Paradise).

In the third generation, the home is property and status that each woman expects to receive from her prospective husband. One of the striking changes in this generation is the women’s demand that their marital home be separate from the husband’s parents’ home. The women perceive their own parents’ home as incarcerating, and they look forward to freedom when they get married, as Siham says: “*I got married because I wanted to get out of my father’s house.…He didn’t let us go out of the house and we love freedom.*” But that freedom proves illusory; the permanent home severs the ties with the community outside.

Siham’s words illustrate the gap between the generations. In the second generation, a woman’s happiness and honor came from staying at home, but in the third generation the home is both desired and perceived as a suffocating place that affords no freedom. The women of the third generation, the granddaughters, dream of their own private home, closed, well furnished, as Siham says: “*I want a home, even a simple one, but it should have curtains, kitchen cabinets, carpets, a bookcase for the children.*” In this generation there is a great emphasis on the material role of the home. This attitude, influenced by global values, is now so deeply rooted that a husband’s inability to give his wife a private home may be a source of tension between them, and even separation. This is in contrast to the second generation, in which the familial and marital relations were the essence and in themselves constituted the home.

In the third generation, building a home is generally the sole responsibility of the husband, and he usually begins before he is married. In some cases, the couple build the home after they are married, and then there is discussion of participation in decision-making, as Siham says: “*We built this home together.”* Siham’s participation in the decisions and the material expenses stands out; as a working woman she could contribute to the joint effort. But she emphasizes that this is not the true permanent home, which exists only in the afterlife: *“This is the [temporary] home of life* [dar el-haia]*, and with our faith we will build our [eternal] home* [dar el-ahra]” in Paradise.

It is important to note that in Bedouin culture, even if the wife participated in building a house, if her husband divorces her, she cannot sue for her part in the home. This situation is very different from that in the first generation, when the home could be erected quickly anywhere and a woman did not have to rely on her husband to obtain a plot of land.

The third-generation woman, especially one who works outside the home, knows that she must also demand a plot that will belong only to the couple—to free her, especially from the tyranny of her mother-in-law—and is not willing to compromise on building the home on the land of other family members, as was the case in the beginning of permanent settlement: “*A home of our own, a plot of our own*” (Lola); “*I told him I do not want this house that is built on your uncle’s plot… I want my own plot*” (Siham). This trend, expressed in the words of Siham and Lola, both of whom are educated and working women, shows the intergenerational difference with regard to land. Whereas in the first and second generations the connection to the land was natural, in the third generation, land has become a commodity.

### Summary

The meaning of home is totally different in each of the three generations. In the first generation, the home symbolizes permanence and women’s creativity, direct connection with the community, and control. In the second generation the home is the family, the relations between the family members and between the husband and wife. In this generation the physical home is perceived as a structure in transition, a structure that is permanent in terms of its location but that is made of temporary materials. In this generation women lose their important role of creating a home and some of the freedom they had in the nomadic period. In the third generation the home symbolizes materialism, acquisitiveness, privacy, and individualism. In the first and second generations women took part in determining the home’s boundaries and in their recognition and preservation, which reduced the breaching of boundaries to a minimum. In contrast, the fact that the young, third generation does not recognize these unclear boundaries has many implications for the lives of young women. One can see in this a change in the perception and meaning of the home in each generation, which reflects a change in values and a social change in the lives of Bedouin women. In the first generation the boundaries of the home were open and penetrable; there were no clear boundaries between the individual space and the public space, so both of them became the women’s living space. In contrast, the boundaries of the home in the third generation are clear and impenetrable; a permanent wall separates the private sphere from the public sphere. The second generation, as I have noted, is in a state of transition characterized by clear but unstable boundaries between the private and the public. Within an iron-sheet home, one can still hear voices from the outside.

## Discussion

Because of state-imposed changes in the Bedouin community’s space, and because Israel’s planning of the Bedouin towns did not take into account the women’s needs, the transition from nomadism to settled life has changed the women’s space. The simple tent was a secure home for the women of the first generation, but the modern, well-furnished home has become a prison for women of the young generation.

In this study we have seen three types of homes: the open and satisfying home, the secure and protecting home, and the modern yet suffocating home.

### The satisfying home (bayt)

The nomadic Bedouin community was far from urban centers and lived in a familiar social and cultural space that provided, especially for the women, a secure and protected space. For the women, this home affords a sense of belonging and intimacy, feelings of strength and control in their space. This is a home that is an inseparable part of the community’s space, allowing active participation in the community, the traditional economy, and, indirectly, local politics. The women identify emotionally with this home (Dam and Eyles 2012).

The internalization of the psychological home creates an emotional protective wall around the women and helps them cope effectively with the changes in their environment. The symbolic connection to the land, to customs, to tradition, and to heritage empowers them and creates strength and stability in their personal identity (Allassad Alhuzail 2013). The older women clearly distinguish between the home they had as nomads and the stone home they live in today. They consider the tent a real home (*bayt*), whereas the current residence is merely a house (*dar*).

The Bedouins, like other indigenous peoples of the world, express nostalgia for the past, for the connection to the land, and for the spirituality in that connection, which granted them power and control. Mitchell (1998) shows how nostalgia helps people cope with distance from the familiar and happy past and the need to navigate an unfamiliar and threatening present. The life stories of the Bedouin women are rich in descriptions of the happy, secure, and unifying *bayt*. Nostalgia and yearning for the past have helped them adjust effectively to the great changes in their lives.

### The secure and protecting home (maskan)

Despite the external threat to this home, it provides protection and security to its inhabitants, a transitional generation that holds the keys to progress for the younger generation. Uprooted from the land to which it was connected, the second generation has had to cope with an imposed new reality, the loss of traditional occupations and the status of having created the home (Allassad Alhuzail 2002; Al-Atawna 2002).

In this generation the women isolated themselves from what was happening in the public sphere and focused on their internal sphere.

It is noteworthy that none of the generations of Bedouin women mention the political processes that Israel’s Arab society has undergone: The Nakba, the expropriation of land, and the demolition of homes never come up in their life stories. This isolation has helped the second generation undergo slow but certain change, cope effectively, and perceive themselves in a way that enables them to push the younger generation toward individual and communal change. Similarly, there is an absence of testimony to the emotional harm engendered by the state’s policy of demolishing Bedouin homes. This silence derives from the women’s emotional connection to the concept of home as family; the personal, defined, and clear identity; and the sense of security in the home and the family that has helped them adapt to the threatening present and see the future through their children’s generation.

### The defined but confused home (dar)

This home symbolizes the temporariness of life. Sometimes called *dar el*-*haia*, it is physically permanent but spiritually temporary. It is built in a period in which the Bedouin community is becoming more religious, the women stay home more and are punctilious about praying so as to merit an eternal spiritual home.

The external form of this generation’s home symbolizes social, economic, and family status. It is built according to a plan, with most of the permits for its construction. It creates a firm separation between the private space and the public space, and it symbolizes privacy, materialism, and secrecy. The quest for happiness in a demanding reality has led the women to enclose themselves in the private sphere of the individual home (Shamir 2008). It symbolizes intimacy, closeness, and affection, as well as distance and the emotional burden in the absence of a spouse or in his massive presence in the home. The idyllic picture Bedouin women draw of the home and married life conceals confusion and vagueness, a home in which a private dynamic—and no longer a tribal dynamic—is manifested, where the increase in the value of privacy has eroded the founding generation’s value of communal life, and particularly that of the women (Dam and Eyles 2012). This is a home that appears to be clearly defined, but is confused, belonging as it does to the generation torn between the will of the individual and the will of the society, a generation at the height of a long process of transition whose many challenges require complex coping and adaptation by Bedouin women (Allassad Alhuzail 2014).

## Summary and conclusions

The geographic space of the Bedouin community has changed substantially in recent decades and with it, the form of housing. The geographic environment during the nomadic period was more suited to the lives of Bedouin women and gave them security and an emotional connection to the land and nature. The imposed transition to urban settlement was planned and implemented without taking their needs into account. The home traditionally represented women’s power and control, but the extent of that power has changed with the change in the home, becoming limited to the private sphere and the nuclear family. Part of the distress engendered by these changes is manifested within the home. In responding to this distress, women can draw on the traditional power of the home as a source of strength, coping, and resilience.
